# The application of metaverse in healthcare

**DOI:** 10.3389/fpubh.2024.1420367

**Published:** 2024-07-29

**Authors:** Yue Wang, Mengying Zhu, Xi Chen, Ruibin Liu, Jinnian Ge, Yuxuan Song, Guilin Yu

**Affiliations:** ^1^Department of General Surgery, Cancer Hospital of China Medical University, Liaoning Cancer Hospital & Institute, Shenyang, China; ^2^Department of Clinical Integration of Traditional Chinese and Western Medicine, Liaoning University of Traditional Chinese Medicine, Shenyang, China; ^3^Department of General Surgery, First Hospital of China Medical University, Shenyang, China; ^4^Pharmaceutical Science, China Medical University-The Queen’s University Belfast Joint College, Shenyang, China

**Keywords:** metaverse, healthcare, virtual reality, artificial intelligence, augmented reality

## Abstract

While metaverse is widely discussed, comprehension of its intricacies remains limited to a select few. Conceptually akin to a three-dimensional embodiment of the Internet, the metaverse facilitates simultaneous existence in both physical and virtual domains. Fundamentally, it embodies a visually immersive virtual environment, striving for authenticity, where individuals engage in real-world activities such as commerce, gaming, social interaction, and leisure pursuits. The global pandemic has accelerated digital innovations across diverse sectors. Beyond strides in telehealth, payment systems, remote monitoring, and secure data exchange, substantial advancements have been achieved in artificial intelligence (AI), virtual reality (VR), augmented reality (AR), and blockchain technologies. Nevertheless, the metaverse, in its nascent stage, continues to evolve, harboring significant potential for revolutionizing healthcare. Through integration with the Internet of Medical Devices, quantum computing, and robotics, the metaverse stands poised to redefine healthcare systems, offering enhancements in surgical precision and therapeutic modalities, thus promising profound transformations within the industry.

## 1 Introduction

In recent years, the rapid advancements in virtual reality (VR) technology have propelled it into a dynamic and rapidly growing field (1). A significant milestone was reached in June 2020 when surgeons at Johns Hopkins University successfully performed their inaugural augmented reality (AR) surgery on a patient. During the first procedure, surgeons incorporated transparent eye displays integrated into headsets to project computed tomography (CT) scan-based images of the patient’s internal anatomy. This innovative approach enabled them to insert six screws and fuse three vertebrae in the spine, effectively alleviating severe back pain (2). Subsequently, a second surgery was conducted to remove a cancerous tumor from the patient’s spine. As we approach the imminent era of the metaverse, digital services are anticipated to revolutionize healthcare, according to the World Economic Forum. The COVID-19 pandemic expedited the widespread adoption of telehealth due to the risks associated with face-to-face interactions, resulting in a significant shift toward remote care.

Telepresence, digital twinning, and the convergence of blockchain technology are three major technological advancements that have the potential to revolutionize healthcare. These concepts could introduce entirely new approaches to delivering care, potentially lowering costs and greatly improving patient outcomes (3). Recognizing the vast possibilities in this uncharted domain, major technology companies have actively entered the landscape, exploring numerous potential applications for the industry. The metaverse embodies the integration and convergence of digital and physical worlds, blending digital and real economies, amalgamating digital and social life, incorporating digital assets into the physical realm, and fusing digital and real identities, thus creating a multidimensional space (4). Various technologies, including high-speed communication networks, the Internet of Things (IoT), AR, VR, cloud computing, edge computing, blockchain, and AI, serve as the foundation for this space. These technologies are driving the transition from the current Internet landscape to the metaverse. The transformation is anchored on eight fundamental technologies, namely extended reality, user interaction, AI, blockchain, computer vision, IoT and robotics, edge and cloud computing, and future mobile networks. Achieving the full realization of a Health metaverse remains a significant challenge in the medical and health domain. Existing platforms still require collaborative efforts from all stakeholders (4). Noteworthy players in the AR and VR market include Google, Microsoft, DAQRI, Psious, Mindmaze, Firsthand Technology, Medical Realities, Atheer, Augmedix, and Oculus VR. These innovative concepts are expected to significantly enhance comprehensive healthcare, revolutionize disease prevention and treatment, and usher in a new era in the industry (5).

**Figure 1:**
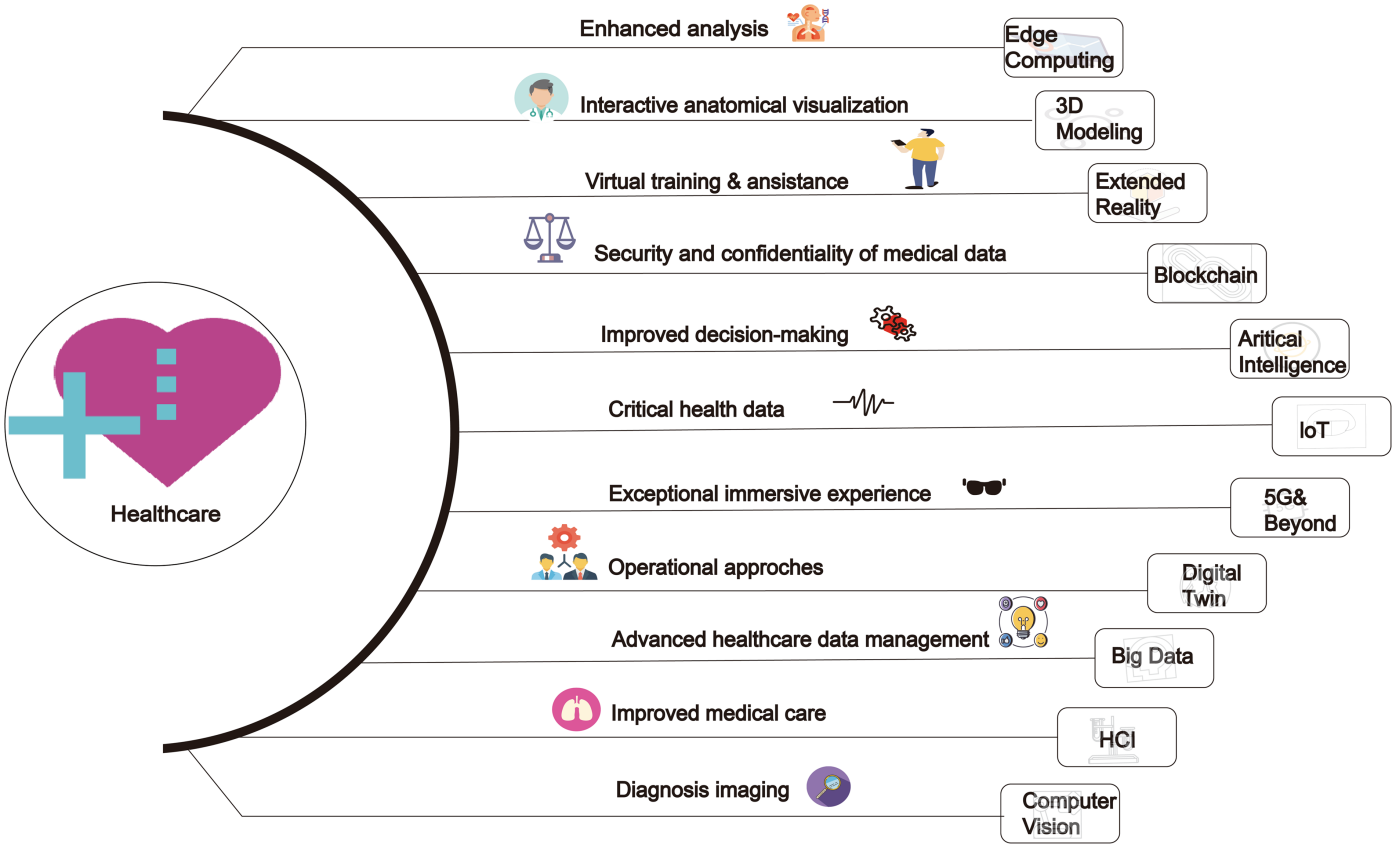
The application of metaverse in healthcare.

In the field of healthcare, the application of metaverse is still in its early stages of development, despite its enormous potential, it also faces several challenges and obstacles. The current technological level has not fully supported the widespread application of metaverse in healthcare. Although VR and AR technologies have made some progress, there are still technical limitations in their application in the medical field, as well as regulatory challenges in healthcare. Additionally, the widespread application of metaverse in healthcare also requires overcoming difficulties in public acceptance and trust in new technologies. Since metaverse is a relatively novel concept, more promotion and education are needed to increase public awareness and acceptance. This review provides an exploration of these issues. This review aims to offer a comprehensive analysis of metaverse in healthcare systems (Summarized in Figure 1).

## 2 Types of the metaverse

This comprehensive review seeks to delineate and classify the four distinct types of the metaverse while elucidating their potential and limitations in educational settings. Specifically, it aims to describe the defining characteristics of each type of metaverse through illustrative examples. Additionally, the review highlights the advantages and applications of incorporating the metaverse into the field of education. Moreover, it engages in a critical examination of the limitations and disadvantages associated with the utilization of the metaverse. Through these analyses, this review aims to provide foundational insights into the concept of the metaverse and its potential applications in the realm of education.

### 2.1 Augmented reality

Augmented reality represents a groundbreaking technology that enriches our perception of the external world by seamlessly integrating location-aware systems and layered networked information into our daily encounters (6). AR interfaces can be classified into three categories: GPS-based, marker-based, and see-through-based (7). Leveraging the capabilities of mobile devices equipped with GPS and Wi-Fi, AR provides contextual information tailored to the user’s location or augments existing information by recognizing markers such as QR codes. Real-time blending of virtual graphics and the physical environment can also be experienced through glasses or lenses. The educational potential of AR has been widely recognized, particularly in areas where direct observation or textual explanations are challenging. It proves invaluable in fields requiring continuous practice, experiential learning, or those associated with high costs and risks (6). As an illustrative example, the Virtuali-Tee developed by Cruscope offers an augmented reality T-shirt that enables students to explore the intricacies of the human body, akin to an anatomy lab experience (8). Another educational application of AR lies in simulation, bridging the gap between abstract visuals and tangible objects by connecting real-world context with virtual elements. In the medical field, numerous examples of AR technology have emerged. Notably, a research team at a Seoul hospital collaborated with university laboratories to develop an augmented reality-based spinal surgery platform. This innovative platform projects real-time images of pedicle screws onto the human body, facilitating spinal fixation procedures (9). Furthermore, this technology serves as the foundation for the development of an effective spinal surgery education program, enabling the implementation of practical training systems. In conclusion, augmented reality blurs the boundaries between the real and virtual worlds, offering transformative opportunities in education and beyond. Its ability to provide enhanced learning experiences and facilitate complex tasks underscores its immense potential for future applications.

### 2.2 Lifelog

A lifelog is a personal record of one’s daily life in a varying amount of detail, for a variety of purposes. The record contains a comprehensive dataset of a human’s activities. The data could be used to increase knowledge about how people live their lives (10). In recent years, some lifelog data has been automatically captured by wearable technology or mobile devices. The sub-field of computer vision that processes and analyses visual data captured by a wearable camera is called “egocentric vision” or egography (11). For example, Steve Mann was the first person to capture continuous physiological data along with a live first-person video from a wearable camera. His experiments with wearable computing and streaming video in the early 1980s led to the formation of Wearable Wireless Webcam (12). Using a wearable camera and wearable display, he invited others to see what he was looking at, as well as to send him live feeds or messages in real-time (13). In 1998 Mann started a community of lifeloggers which has grown to more than 20,000 members. In 1996, Jennifer Ringley started JenniCam, broadcasting photographs from a webcam in her college bedroom every 15 s; the site was turned off in 2003 (14). The lifelog DotComGuy ran throughout 2000, when Mitch Maddox lived the entire year without leaving his house (15). After Joi Ito’s discussion of Moblogging, which involves web publishing from a mobile device (16), came Gordon Bell’s MyLifeBits (17), an experiment in digital storage of a person’s lifetime, including full-text search, text/audio annotations, and hyperlinks.

In recent years, with the advent of smartphones and similar devices, lifelogging became much more accessible. For instance, UbiqLog (18) and Experience Explorer (19) employ mobile sensing to perform life logging, while other lifelogging devices, like the Autographer, use a combination of visual sensors and GPS tracking to simultaneously document one’s location and what one can see. Lifelogging was popularized by the mobile app Foursquare, which had users “check in” as a way of sharing and saving their location; this later evolved into the popular lifelogging app, Swarm.

### 2.3 Mirror world

The mirror world represents a simulated version of the physical realm, characterized by an informationally enhanced virtual model or a “reflection” of reality. It serves as a metaverse where the real world’s appearance, information, and structure are transferred into virtual reality, akin to the reflection seen in a mirror (20). However, it is more appropriate to describe these systems as enabling “efficient expansion” rather than mere reproductions of reality (21). In this virtual domain, all real-world activities can be conducted through internet platforms or mobile applications, offering convenience and efficiency to daily life (22). Notably, mirror world metaverses have found application in education through the creation of “digital laboratories” and “virtual educational spaces” across various mirror world platforms. In essence, the mirror world acts as a bridge between the tangible and intangible, enhancing our interaction with the world around us (23). By seamlessly integrating the convenience of digital platforms with the richness of our physical surroundings, it opens up new possibilities for education and beyond (24).

### 2.4 Virtual reality

VR represents a metaverse that simulates the inner world, leveraging sophisticated 3D graphics, avatars, and instant communication tools (25). As users immerse themselves in this virtual realm, they experience a profound sense of presence. VR can be viewed as the opposite end of the spectrum from mixed reality and augmented reality. However, this technology relies on our eyes’ working principle to present flat images in three dimensions. It also serves as an internet-based 3D space where multiple users can simultaneously access and participate by creating expressive avatars that reflect their true selves. VR offers an unparalleled opportunity to explore the intricacies of the human mind, enabling us to navigate the inner world like never before. By leveraging cutting-edge technologies, it opens up new vistas for communication, education, and entertainment, paving the way for a future where the boundaries between the real and virtual worlds continue to blur.

In this VR metaverse, the spatial configurations, cultural contexts, characters, and institutions are uniquely crafted, diverging from their real-world counterparts (26). Through user-controlled avatars, individuals navigate virtual spaces inhabited by AI characters, fostering interactions with fellow players and pursuing designated objectives. Narrowly construed, VR, also known as the metaverse, encompasses scenarios where physical actions, tactile sensations, and routine economic activities unfold within the virtual realm (27). For instance, Zepeto and Roblox exemplify VR platform. Zepeto, a recently introduced 3D avatar-based interactive service, and Roblox, a versatile platform enabling user-generated virtual worlds and games, epitomize this evolving landscape. Zepeto, an augmented reality avatar service under Naver Z, stands as a prominent metaverse platform in Korea, leveraging facial recognition, augmented reality, and 3D technology to facilitate user communication and diverse virtual experiences since its launch in 2018. Notably, Roblox, featuring the use of virtual currency and a fully realized economic ecosystem. Its hallmark lies in users’ ability to construct and partake in self-authored VR games using Lego-shaped avatars or to immerse themselves in experiences crafted by others. When capturing or uploading an image on a smartphone, AI technology generates a character resembling the user, allowing for extensive customization of skin tone, facial features, height, expression, gestures, and fashion preferences (28). This interactive experience incorporates social networking service (SNS) functionalities, facilitating user connections to follow others and engage in text or voice-based communication. Within this VR framework, diverse activities, including gaming and educational role-playing, can be seamlessly conducted across multiple maps. For instance, educators can opt for a classroom map, create a virtual room, invite students, and foster interactive exchanges through voice or messaging within the designated classroom environment.

## 3 Medical education

Transformation awaits medical education and training through the integration of AR and VR. With the ability to virtually immerse themselves in the human body, students gain comprehensive perspectives, enabling them to replicate real-life treatments (29). AR also offers hands-on learning opportunities by simulating patient and surgical interactions, empowering medical interns to envision and practice novel techniques (30). Furthermore, the development of more realistic experiences based on actual surgeries allows students to engage in surgery as if they were the surgeons themselves. This immersive approach to learning not only rewards success but also employs data analytics to target precision education. Traditional medical schools face resource limitations when it comes to practical surgical training due to the expenses associated with cadaver surgeries and their impact on students’ tuition fees. However, incorporating VR in medical education enables trainees to undergo intensive surgical instruction within a simulated environment at a significantly reduced cost, surpassing mere knowledge transmission. Metaverse-based healthcare training necessitates additional technological advancements for advanced hand skills and interactions. For instance, surgical interventions require precise mastery of human anatomy and dexterity in handling equipment, which can be facilitated through appropriate tracking devices or software. Leveraging technology in the metaverse can also aid in complex surgical procedures as doctors strive for higher success rates. By utilizing data collected from a patient’s digital twin, doctors can estimate recovery periods, anticipate potential difficulties, and plan necessary therapies as part of a preventive strategy (21). Instructors play a crucial role in providing high-quality data to support virtual programs that simulate on-site nursing competencies. Learners should feel no difference in clinical therapy when transitioning from the metaverse context to a real clinical field experience program. Such advancements hold significant promise for improving patient care in the long run.

Here are examples of AR and VR in the application of medical education (31–34).

1. VR in Sexual Disorders Treatment: The document describes a randomized controlled trial using an avatar-based sexual therapy program on a metaverse platform for treating female orgasm disorder (FOD). This program, conducted in the virtual environment of Second Life, included 12 weekly online sessions where participants received cognitive-behavioral therapy (CBT) and acceptance and commitment therapy (ACT). The results showed significant improvements in sexual satisfaction, reduced sex guilt, and lower anxiety among participants.
2. VR for Autism Spectrum Disorders (ASD): A metaverse-based social skills training program was developed using the Roblox platform. This program, which included four 1-h sessions per week for 4 weeks, focused on improving social interaction abilities among children with ASD. The sessions included theoretical knowledge, group activities, and practical exercises in the metaverse, leading to significant gains in social skills and mental health outcomes.
3. AR in Anatomy Education: Although not explicitly detailed in the extracted text, similar applications in the metaverse often involve using AR to overlay digital anatomical structures onto physical bodies, allowing medical students to visualize and interact with these structures in real-time. This method enhances their understanding of human anatomy without the need for physical cadavers.
4. 3D Human Replicas and Virtual Dissection: The document discusses the use of 3D human replicas and virtual dissection to enhance medical education. This allows students to interact with and manipulate digital anatomical structures, providing a deeper understanding of complex anatomical features and structural pathologies. For example, tools like the Holonomy Software enable the visualization of human anatomy without the need for cadavers, significantly enhancing the learning experience.
5. Virtual Laboratories and Simulations: Virtual science labs provide a secure environment for hands-on experimentation. These simulations allow students to manipulate objects, mix substances, and observe reactions, aiding in the understanding of complex concepts such as blood flow behavior in vessels by experimenting with pressure-volume curves in cardiac cycles and blood circulation.
6. *In-situ* Learning and Authentic Assessment: The metaverse situates medical students in authentic environments like hospitals, adding realism to their learning. Simulated workplaces prepare students for future professional endeavors, and virtual field trips to simulated disaster control sites or common injury scenarios offer low-cost solutions for hands-on learning experiences. This method focuses on evaluating students’ skills in meaningful, real-world contexts.
7. Interdisciplinary Learning: The metaverse allows for the seamless integration of multidisciplinary information, facilitating an understanding of complex medical concepts by combining disciplines such as anatomy, physiology, pathology, and pharmacology. Virtual ‘stations’ and dynamic group formations support discussions, problem-based learning, and team-based learning sessions without the limitations of physical classrooms.

## 4 Technical detail and accessibility

### 4.1 Software requirements

#### 4.1.1 VR and AR platforms

1. Unity and Unreal Engine: These are powerful game development engines crucial for creating immersive healthcare simulations. They offer advanced 3D rendering capabilities for highly detailed and realistic 3D models of human anatomy, medical instruments, and clinical environments. These engines also feature advanced physics simulation for realistic modeling of physical interactions, vital for surgical procedures and emergency response drills. Their support for highly interactive environments enables hands-on training, allowing medical students to practice procedures, make decisions, and receive feedback in real-time. Additionally, their cross-platform support ensures accessibility across various devices, making educational content available to a broader audience.
2. AR/VR SDKs: R/VR Software Development Kits (SDKs) such as Oculus SDK, ARKit, and ARCore provide essential tools for creating applications tailored to specific AR/VR hardware, ensuring compatibility and optimized performance for high-quality educational experiences in healthcare. The Oculus SDK, developed by Facebook, supports creating immersive VR simulations with features like head tracking, hand tracking, and spatial audio, crucial for realistic medical training. Apple’s ARKit enables the development of augmented reality applications on iOS devices, allowing medical students to overlay digital information onto the real world, enhancing their learning by visualizing anatomical structures and simulating procedures. Similarly, Google’s ARCore provides tools for building AR applications on Android devices, offering motion tracking, environmental understanding, and light estimation, thus creating interactive AR experiences that enhance medical education by integrating virtual elements into physical environments.

#### 4.1.2 Healthcare-specific applications

1. Telemedicine Platforms: Telemedicine platforms like XRHealth are becoming increasingly vital in modern healthcare by offering remote consultations, monitoring, and therapy sessions through VR. XRHealth enables doctors to conduct virtual consultations, enhancing communication and understanding between doctors and patients. The platform supports remote monitoring of health conditions, allowing patients to engage in therapeutic exercises and rehabilitation programs under supervision. VR-based therapy sessions address various conditions, providing interactive and engaging experiences that promote better adherence to treatment protocols. By leveraging VR, XRHealth makes healthcare services more accessible to patients in remote or underserved areas, reducing the need for travel and enabling timely medical intervention.
2. Simulation Software: Simulation software platforms like SimX and Osso VR revolutionize medical training by providing detailed, risk-free virtual simulations. SimX offers realistic training scenarios, collaborative learning through multiplayer functionality, and customizable scenarios for targeted skill development. Osso VR focuses on surgical training with interactive simulations, step-by-step guidance, real-time feedback, and assessment tools for certification. Both platforms enhance skill acquisition through interactive practice and provide scalable, accessible training solutions, making high-quality medical education available to a broader audience, especially in regions with limited access to advanced facilities.

#### 4.1.3 Data management and security

1. EHR Integration: EHR integration within the metaverse, using systems like Epic and Cerner, is crucial for providing seamless access to comprehensive patient records, ensuring informed and accurate medical care. This integration offers healthcare professionals real-time access to medical histories, streamlines workflows by reducing manual data entry, and enhances care coordination among different providers. Patients also benefit by accessing their health records, tracking progress, and communicating with healthcare providers more efficiently, leading to better health outcomes and proactive chronic condition management.
2. Blockchain Technology: Utilizing blockchain technology for secure, immutable record-keeping enhances data security and patient privacy within the metaverse by providing a decentralized and transparent system that ensures the integrity of medical records. Blockchain’s immutable record-keeping prevents data alteration or deletion, maintaining accurate medical histories and accountability. Advanced cryptographic techniques secure sensitive information from unauthorized access and cyberattacks. Decentralized data storage reduces the risk of data breaches, while smart contracts enable patients to control access to their records, ensuring compliance with data protection regulations like GDPR and HIPAA. Blockchain’s transparency allows for traceable transactions, ensuring accountability and enabling audits to verify proper data handling.

### 4.2 Hardware requirements

#### 4.2.1 VR/AR headsets

1. High-End Devices: High-end VR headsets are essential for delivering the detailed and high-fidelity simulations required in medical training. The Oculus Rift, known for its high-resolution displays and precise motion tracking, provides an immersive experience crucial for detailed anatomical studies and complex surgical simulations. The HTC Vive offers a wide field of view and high precision in motion tracking, making it suitable for applications that require accurate representation of movements, such as physical therapy training and interactive diagnostic procedures. The Valve Index, with its advanced display technology and finger-tracking capabilities, is ideal for simulations requiring intricate hand movements and interactions, such as virtual dissections and surgical practice.
2. Standalone Devices: Standalone VR headsets like the Oculus Quest 2 provide a wireless experience with built-in processing power, eliminating the need for external computers or cables. Its portability makes it suitable for various healthcare settings, from remote consultations to on-site training sessions, and is particularly beneficial for rural and underserved areas with limited access to high-end equipment.

#### 4.2.2 Peripheral devices

1. Haptic Feedback Devices: Haptic feedback devices enhance the realism of virtual simulations by allowing users to feel tactile sensations. HaptX Gloves provide detailed tactile feedback and resistance, enabling users to feel textures and forces when interacting with virtual objects, which is crucial for surgical training. The Teslasuit offers full-body haptic feedback and motion capture, enhancing the realism of simulations involving physical therapy and rehabilitation exercises by precisely replicating real-world sensations, thus making training more effective.
2. Motion Tracking Systems: Accurate motion tracking is essential for applications requiring detailed analysis of body movements. Microsoft Kinect’s advanced motion capture capabilities track precise movements, making it suitable for physical therapy applications where monitoring the patient’s range of motion and ensuring correct exercise techniques are critical. OptiTrack offers high-precision motion tracking for surgical training and ergonomic studies, providing detailed data on movement patterns and helping improve procedural accuracy.

#### 4.2.3 Computing power

1. High-Performance Workstations and Servers: High-performance workstations and servers are essential for effective VR/AR medical simulations, providing the robust computing resources needed for rendering complex simulations, managing large datasets, and ensuring seamless user experiences. High-performance workstations feature NVIDIA RTX Series GPUs for high-fidelity rendering, multi-core processors like Intel Xeon or AMD Ryzen Threadripper for computational power, large memory capacities (32GB+ RAM) for data-intensive tasks, and high-speed NVMe SSDs for quick data access. High-performance servers support collaborative and large-scale simulations through clustered computing, scalable architecture, high-throughput networking (such as 10GbE), and advanced data management solutions like RAID arrays and NAS systems, ensuring efficient and reliable data handling and real-time interactions.

### 4.3 Network requirements

#### 4.3.1 Bandwidth and latency

1. High-Speed Internet: Reliable high-speed internet is a fundamental necessity for VR/AR applications in healthcare, enabling quick and efficient data transmission. Fiber optic connections deliver the required bandwidth, offering speeds exceeding 1 Gbps to support the seamless streaming and downloading of large medical datasets, high-resolution 3D models, and real-time video feeds. In remote consultations, high-speed internet ensures uninterrupted communication with clear audio and video quality for both healthcare providers and patients, contributing to stable and reliable connectivity critical for maintaining the effectiveness of VR/AR-based medical applications during critical procedures and training sessions.
2. Low Latency Networks: Low latency networks are crucial for smooth and responsive real-time VR/AR applications in healthcare. The emergence of 5G technology has significantly reduced network latency, offering ultra-low latency as low as 1 millisecond. This low latency is especially important in healthcare scenarios like remote surgery, where real-time feedback and precise control are critical. Surgeons using VR equipment to perform procedures remotely can rely on the quick response time provided by 5G networks to make accurate and timely movements, minimizing the risk of errors. Additionally, low latency networks enhance the overall user experience by ensuring that VR/AR interactions are seamless and responsive, particularly in immersive training simulations where delays can disrupt the learning process and reduce the realism of the experience.

#### 4.3.2 Cloud and edge computing

1. Cloud Services: Cloud services are essential for supporting large-scale metaverse healthcare applications due to their scalability and flexibility. Providers like AWS, Google Cloud, and Microsoft Azure offer on-demand computing power and storage, enabling healthcare applications to scale resources based on demand. This scalability is crucial for handling peak usage times, such as during virtual training sessions or remote consultations. Cloud services also provide robust data storage solutions, ensuring the secure storage and easy accessibility of medical datasets, patient records, and simulation data. Advanced data management tools offered by cloud providers help organize, retrieve, and analyze data efficiently, facilitating improved decision-making and patient care. Additionally, utilizing cloud services can be more cost-effective than maintaining on-premises infrastructure, allowing healthcare organizations to reduce capital expenditure and only pay for the resources they use, making cloud computing a financially viable option for deploying VR/AR healthcare applications.
2. Edge Computing: Edge computing is pivotal for enhancing the performance of VR/AR applications in healthcare by bringing computing resources closer to the data source. This approach reduces latency, improving the responsiveness of time-sensitive medical applications and ensuring real-time processing for effective experiences like VR-based physical therapy sessions. Additionally, edge computing offloads processing tasks from central cloud servers to local edge devices, thereby enhancing overall performance and reducing the load on the network, particularly beneficial for real-time processing and high-speed data transfer in applications such as surgical simulations and diagnostic tools. Furthermore, processing data at the edge can enhance security and privacy by minimizing sensitive information transmitted over the network, reducing the risk of data breaches and safeguarding patient information.

### 4.4 Accessibility considerations

#### 4.4.1 User interface and experience

1. Intuitive Design: Designing intuitive and accessible user interfaces is crucial for effective use of metaverse applications in healthcare by both professionals and patients, regardless of their technical expertise. This involves implementing simple and clear navigation paths, a clean layout, organized menus, and consistent design language. Additionally, using large icons and text improves readability and usability, especially for users with visual impairments or smaller screens. Integration of voice instructions can further enhance the user experience, guiding users through tasks and procedures, particularly benefiting those dealing with complex workflows or preferring auditory learning.
2. Voice and Gesture Controls: Implementing voice commands and gesture-based controls in metaverse applications significantly enhances accessibility for users with physical disabilities. Voice recognition technology allows users to perform tasks through verbal instructions, reducing the need for manual input. This benefits patients with limited mobility and healthcare professionals who require hands-free operation during procedures. Gesture recognition enables interaction with the application using hand and body movements, providing an alternative input method for individuals unable to use traditional devices like a mouse or keyboard. This intuitive approach enhances the user experience, offering a natural and seamless interaction method.

#### 4.4.2 Affordability and training

1. Cost-Effective Solutions: Developing cost-effective hardware and software solutions is crucial for making advanced healthcare services accessible to a broader population, especially in low-resource settings. Affordable VR/AR headsets and peripheral devices can increase adoption rates among healthcare providers and patients, with collaborations aimed at producing budget-friendly yet high-quality options. Utilizing open-source software for metaverse applications reduces costs and allows for customizable solutions that meet specific needs, while also fostering community contributions that drive continuous improvements and innovation.
2. Training and Support: Providing comprehensive training and support is essential for the successful adoption and utilization of metaverse applications in healthcare. Detailed tutorials and user manuals should be available to help users navigate and effectively use the applications, covering basic operations, advanced features, and troubleshooting tips. Conducting workshops and webinars allows for hands-on training and live demonstrations, where users can ask questions and receive real-time assistance. These sessions can be tailored to different user groups, such as medical staff, administrative personnel, and patients. Ongoing support services, including help desks, chatbots, and customer service representatives, ensure that users have access to assistance when needed. Timely and effective support enhances user satisfaction and confidence in using the applications.

#### 4.4.3 Regulatory compliance

1. Standards and Guidelines: Adhering to healthcare standards and guidelines is crucial for ensuring the safety and efficacy of metaverse applications in healthcare. Compliance with regulatory bodies such as the FDA and EMA involves rigorous testing, validation, and certification processes to maintain safety and effectiveness. Additionally, adherence to data privacy laws like HIPAA and GDPR requires robust data encryption, secure authentication methods, and access control measures to protect patient information. Furthermore, compliance with interoperability standards set by organizations like HL7 and FHIR ensures seamless data exchange with other healthcare systems, enabling comprehensive patient care and continuity of information across platforms and providers.

#### 4.4.4 Inclusivity and accessibility features

1. Adaptive Technologies: Incorporating adaptive technologies into metaverse applications is essential for ensuring accessibility and inclusivity for users with varying abilities, thereby creating equitable and effective healthcare solutions. Integrating screen reader compatibility helps visually impaired users navigate the applications by converting text and visual elements into speech or Braille. Adjustable font sizes allow users to customize text display for better readability, while adjustable color contrast settings assist those with color blindness or low vision by enhancing element distinction and reducing eye strain. Additionally, implementing voice feedback provides auditory cues and confirmations, aiding users with visual impairments in their interactions and navigation within the applications.
2. Multi-Language Support: Offering multi-language support in metaverse healthcare applications is crucial for promoting inclusivity and global accessibility, enabling non-English speakers to benefit from the services. Users should have the option to select their preferred language from a comprehensive list during initial setup or within the application’s settings. Localized content, which adapts to cultural nuances and regional medical terminology, ensures that information is relevant and understandable. In regions with multiple predominant languages, bilingual user interfaces allow seamless switching between languages, enhancing user experience. Additionally, incorporating voice recognition and synthesis capabilities for multiple languages enables users to interact through voice commands and receive auditory feedback in their native language, improving usability for those who may struggle with reading or typing in a non-native language.

## 5 Prospects

Over the next 5 years, we can expect significant developments in the application of the metaverse in healthcare. As technology continues to advance, the metaverse holds immense potential to revolutionize various aspects of healthcare delivery and patient care. We anticipate several key developments: (1) Enhanced Virtual Healthcare Experiences: The metaverse will enable the creation of immersive virtual environments where patients can receive healthcare services remotely. This includes virtual clinics, telemedicine consultations, and remote monitoring of vital signs and health data. (2) Training and Education: Healthcare professionals will increasingly use the metaverse for training simulations, medical education, and skill development. VR and AR technologies will provide realistic training scenarios and hands-on experiences for medical students, nurses, and other healthcare professionals. (3) Virtual Therapy and Rehabilitation: The metaverse will play a significant role in mental health treatment and physical rehabilitation. Virtual reality therapy will offer immersive environments for exposure therapy, relaxation exercises, and cognitive-behavioral interventions. Additionally, virtual rehabilitation programs will assist patients in recovering from injuries or surgeries through interactive exercises and simulations. (4) Collaborative Healthcare Platforms: The metaverse will facilitate collaboration among healthcare providers, researchers, and patients. Virtual meeting spaces and collaborative platforms will enable multidisciplinary teams to discuss cases, share knowledge, and collaborate on research projects regardless of geographic location. (5) Personalized Healthcare Solutions: With advances in AI and data analytics, the metaverse will support personalized healthcare solutions tailored to individual patient needs. AI-powered virtual assistants will provide personalized health recommendations, medication reminders, and lifestyle coaching based on real-time health data and patient preferences. Overall, the metaverse holds immense promise for transforming healthcare delivery, improving patient outcomes, and advancing medical research. However, challenges such as data privacy, security, and accessibility must be addressed to realize the full potential of this technology in healthcare.

Although the application of metaverse in healthcare holds promising prospects, there are several challenges that need to be addressed. These challenges include but are not limited to the following aspects: (1) Technological Maturity and Stability: Despite significant advancements in metaverse technology, there is still a need for more mature and stable technical support, especially in areas such as remote diagnosis, surgical simulation, and virtual therapy. Ensuring the stability and reliability of the metaverse platform is crucial to safeguarding patient safety and healthcare quality. (2) Data Privacy and Security: As the use of metaverse in healthcare increases, there are higher requirements for the privacy and security of patients’ health data. Strict data protection measures and access control mechanisms need to be established to prevent unauthorized access and misuse of patient data, as well as encryption and protection of data transmission and storage processes. (3) User Experience and Accessibility: Achieving widespread adoption of metaverse in healthcare requires consideration of user experience and accessibility issues. It is essential to ensure that the metaverse platform has a user-friendly interface, simple operation, and provide training and support services to help healthcare professionals and patients quickly adapt to new workflows and communication methods. (4) Legal Regulations and Regulatory Policies: With the continuous expansion of metaverse applications in healthcare, it is necessary to establish corresponding legal regulations and regulatory policies to regulate the use and operation of metaverse platforms. This includes the formulation and enforcement of laws on data privacy protection, standards for medical virtual reality technology, and regulations on medical device supervision. (5) Cost and Resource Investment: The application of metaverse requires substantial financial and resource investment, involving hardware equipment, software development, talent training, and other aspects. When healthcare institutions and enterprises consider adopting metaverse technology, they need to comprehensively consider cost-effectiveness, long-term benefits, as well as compatibility and integration with existing medical systems.

In conclusion, while the application of metaverse in healthcare offers vast prospects, overcoming numerous challenges and obstacles is essential. Only through continuous innovation and collaboration can we fully leverage the advantages of metaverse technology to achieve transformation and progress in the healthcare field.

## Author contributions

YW: Conceptualization, Writing – original draft, Writing – review & editing. MZ: Data curation, Investigation, Writing – original draft. XC: Validation, Writing – review & editing. RL: Investigation, Writing – original draft. JG: Investigation, Resources, Supervision, Writing – original draft, Writing – review & editing. YS: Investigation, Writing – review & editing. GY: Validation, Writing – original draft, Writing – review & editing.
